# Epiretinal Membrane after Laser In Situ Keratomileusis

**DOI:** 10.1155/2013/610302

**Published:** 2013-04-04

**Authors:** Miguel Paciuc-Beja, Gerardo Garcia, Jose Dalma, Hugo Quiroz-Mercado

**Affiliations:** ^1^Denver Health Medical Center, University of Colorado, 11981 E Lake Cir, Greenwood Village, CO 80111, USA; ^2^Asociacion para Evitar la Ceguera en Mexico, 04030 Mexico City, DF, Mexico

## Abstract

Multiple posterior segment complications can occur after LASIK. Posterior vitreous detachment, macular holes, retinal hemorrhages, retinal detachment, and several other complications have been described. A case of posterior vitreous detachment with epiretinal membrane in a young adult after LASIK is reported. LASIK surgeons must be aware of the possibility of posterior segment complications after surgery.

## 1. Introduction

Complications after laser in situ keratomileusis (LASIK) are usually reported by refractive surgeons. They are often related to refractive outcome, or to cornea and anterior segment structures. Posterior segment complications are diagnosed and treated by vitreoretinal surgeons, and sometimes the association with previous LASIK surgery is missed. 

Posterior segment complications of LASIK have been reviewed by Mirshahi and Baatz [1], and although rare, there are case reports on posterior vitreous detachment, rhegmatogenous retinal detachment, choroidal neovascularization and macular hemorrhage, macular hole and cystoid macular edema, and visual field defects and vascular events. 

## 2. Case Report

**Figure 1:**
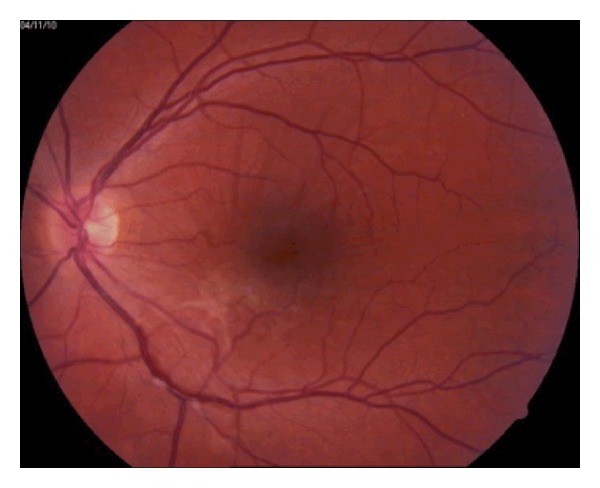
Color fundus photograph of the left eye. The macular region shows macular folds and distortion of the normal architecture of the juxtafoveal vessels. Being nasal and inferior to the fovea, there is evidence of an epiretinal fibrous tissue, which is likely due to the presence of an epiretinal membrane.

**Figure 2:**
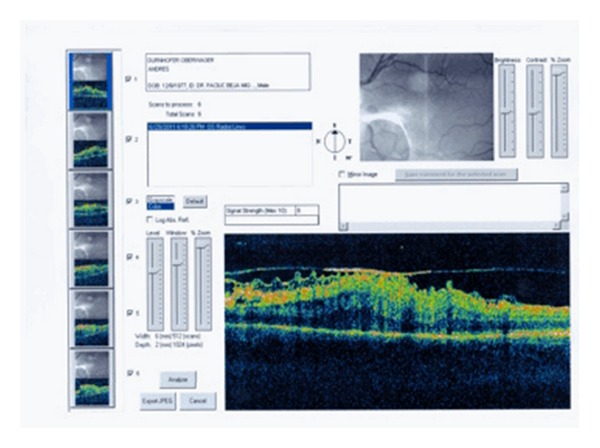
Time domain optical coherence tomography from the left eye shows a complete distortion of the internal and external architecture of the retina, being nasal and inferior to the fovea. There is a highly reflective membrane over the retina tissue, which makes contact with the retina in the central part of the projection. The nerve fiber layer shows a characteristic “Saw” configuration. There is a substantial increase in the retinal thickness.

A 29-year-old male patient was willing to have refractive surgery. Cycloplegic refraction was of −2.50 sph in both eyes. Corneal pachymetry was 549 *μ*m in OD and 530 *μ*m in OS. Dilated funduscopy was unremarkable. LASIK was performed using a Hansatome microkeratome with a 160 *μ*m flap, using a VISX 4 excimer laser. Visual acuity was 20/20 after 6 months. Dilated funduscopy revealed posterior vitreous detachment in both eyes. Eighteen months after surgery, visual acuity was 20/20 in both eyes, but the patient complained of metamorphopsia in OS. Funduscopy revealed an epiretinal membrane in OS, with thin macular folds (Figure 1). Stratus OCT of the macula showed a hyperreflective line that was partially in contact with the retinal surface, folds in the interior layers of the retina, retinal thickening, and distortion of the normal retinal architecture (Figure 2). 

## 3. Discussion

Several posterior segment complications have been described after LASIK [1–3]. Complications range from retinal tears, retinal detachments, choroidal neovascularization, subretinal hemorrhage, and macular hole. Incidence of complications has been estimated to be from 0.06% [2] to 0.36% [4].

The relationship between the LASIK procedure and vitreoretinal complications is difficult to establish, since such complications occur with a higher incidence in patients with high myopia, being the majority of patients undergoing refractive surgery. There is evidence, however, that suggests that the incidence of vitreoretinal complications is higher in these patients compared to the expected incidence in patients with similar characteristics in which this procedure is not performed. There is also the fact that vitreoretinal complications observed share a common pathophysiology, which is posterior vitreous detachment (PVD) [4], and that there is a cause-effect relationship between LASIK surgery and PVD [5, 6]. In a comparative study performed by Luna et al. [5], modifications to the vitreous body in 100 eyes of 50 patients using ocular ultrasonography before and after myopic microkeratome-assisted LASIK were analyzed, and the overall percentage of postoperative PVD was found to be 14%. Further evidence has been provided by Mirshahi et al. [6], which found an incidence of postoperative PVD of 9.5%. In another comparative study performed by Gavrilov et al. [7], B-scan ultrasound was performed before and after LASIK surgery in 31 eyes in which femtosecond laser was used to create the flap, showing that 48 hours after the procedure, 16% of the eyes had induction of PVD. 

Several hypotheses have been offered to explain the relationship between LASIK surgery and the occurrence of PVD, and therefore of posterior segment complications. It has been suggested that post-LASIK PVD might be caused by either globe deformation secondary to increased intraocular pressure with the suction ring or the shockwave of the excimer laser [1]. 

Globe deformation could occur theoretically when the suction ring induces an increase in intraocular pressure, which could rise up to 90 mmHg [8]. The suction and the increase in pressure could elongate the eye along the anteroposterior axis, which in turn could cause a contraction in the horizontal axis. This combination of events may push the lens anteriorly and cause vitreoretinal traction at the vitreous base and the posterior pole and facilitate PVD. 

The other theoretical factor that could induce PVD after LASIK is the trauma caused by the excimer laser shockwave. This has been measured by Krueger et al. [9] in human and porcine eyes, registering stress wave amplitudes in the former of up to 100 atm, 6.2 to 7.3 mm behind the endothelium (corresponding approximately to the posterior part of the lens or the anterior aspect of the vitreous). At the retina level, 23 mm behind the endothelium, the stress wave amplitude fell to approximately 10 atm, which seems to be insufficient to cause a significant retinal lesion.

Posterior vitreous detachment (PVD) has been widely associated with the occurrence of epiretinal membranes. Separation of the vitreous from the inner retinal surface is usually due to the aging process and is more common in myopic eyes. It may also occur as a consequence of trauma or inflammation at any age. PVD has been shown to cause small breaks in the internal limiting membrane through which glial cells may grow and proliferate on the retinal surface [10]. PVD may also be implicated in the formation of preretinal fibrosis by releasing cells from the retinal pigment epithelium through small retinal breaks. Whether in our case PVD was a result of LASIK or not, it is hard to establish. 

In conclusion, LASIK surgery, although safe, is not free of complications. 

It is possible that some patients that had LASIK had a PVD and an asymptomatic, undetected epiretinal membrane.

This case represents an unusual complication of a common surgical procedure.

Patients should be warned about possible posterior segment side effects that may be related to the procedure itself.
